# Effect of early neuroendovascular team involvement in acute stroke protocol: a retrospective study

**DOI:** 10.3389/fneur.2025.1568572

**Published:** 2025-06-17

**Authors:** Hitoshi Mori, Masahiro Kashiura, Ichiro Suzuki, Fumiko Ono, Yuya Yoshimura, Takashi Moriya

**Affiliations:** ^1^Department of Emergency and Critical Care Medicine, Hachinohe City Hospital, Hachinohe, Japan; ^2^Department of Emergency and Critical Care Medicine, Saitama Medical Center, Jichi Medical University, Saitama, Japan; ^3^Department of Neurosurgery, Hachinohe City Hospital, Hachinohe, Japan

**Keywords:** stroke team protocol, tissue plasminogen activator, endovascular therapy, neurological outcomes, workflow optimization, door-to-treatment time

## Abstract

**Introduction:**

Acute ischemic stroke (AIS) is a leading cause of morbidity and mortality, with outcomes dependent on timely treatment. Tissue plasminogen activator (tPA) and endovascular therapy (EVT) improve outcomes, but delays reduce their efficacy. This study introduced a protocol featuring early participation of neuroendovascular interventionists and evaluated its association with treatment times and outcomes compared with conventional management.

**Methods:**

This single-center retrospective study included patients with AIS transported to emergency room (ER) who received tPA or EVT between January 2010 and December 2022. Under the protocol, the stroke team—including neuroendovascular interventionists, who made the final decision on tPA and EVT—was activated by the emergency physician when stroke was suspected based on pre-hospital information. The stroke team was not activated if neuroendovascular interventionists were engaged in other procedures or if the ER physician suspected a non-stroke diagnosis. Upon arrival, the team commenced care, with neuroendovascular interventionists reviewing imaging and determining treatment strategies. Patients were categorized into protocol and conventional groups based on management under the new protocol or standard care. The primary outcome was a favorable neurological outcome, defined as a modified Rankin Scale (mRS) score of 0–2 at discharge. Secondary outcomes included time metrics for initiation of tPA and/or EVT. Logistic regression analysis estimated the effects of the protocol, adjusting for confounders, including age, sex, baseline National Institutes of Health Stroke Scale score, and pre-hospital factors. Secondary outcomes were assessed using multiple linear regression.

**Results:**

This study analyzed 501 patients, with 313 in the protocol group and 188 in the conventional group. Favorable neurological outcomes at discharge (mRS 0–2) were more frequent in the protocol group (44.4% vs. 31.9%; adjusted odds ratio: 2.92, 95% confidence interval [CI]: 1.83–4.66). The protocol group also showed shorter door-to-imaging time (−8.3 min), door-to-needle time (−55.9 min), door-to-puncture time (−59.8 min), and door-to-recanalization time (−73.7 min).

**Conclusion:**

Early engagement of neuroendovascular specialists in the emergency pathway was associated with faster treatment initiation and a higher likelihood of favorable functional status at discharge in this retrospective cohort. Because residual confounding and temporal changes in stroke care cannot be excluded, prospective validation in other settings is warranted.

## 1 Introduction

Acute ischemic stroke (AIS) caused by large vessel occlusion (LVO) is associated with high morbidity and mortality, with outcomes largely dependent on timely recanalization to save the penumbra—the ischemic and viable brain tissue at risk of infarction (1). Established therapies such as tissue plasminogen activator (tPA) (2, 3) and endovascular therapy (EVT) (4, 5) significantly improve recanalization rates and neurological outcomes. Early and successful recanalization after symptom onset is critical for achieving optimal outcomes (6). Preventing logistical barriers—such as delays to treatment and clinical barriers—such as symptomatic intracranial hemorrhage—contributes to improved prognosis (7).

Establishing in-hospital protocols and optimizing workflows for the initial management of AIS can reduce the time required for tPA administration and EVT (8–10), ultimately improving neurological outcomes (11). Despite efforts to optimize workflows, reducing the door-to-puncture time to the recommended 60 min remains challenging (12), with studies showing that only approximately half of eligible patients achieve this target (13).

To address these delays, our institution implemented a new protocol aimed at expediting the decision-making and treatment processes for patients with AIS. This protocol emphasizes the early involvement of interventional neuroradiologists, streamlining care from patient arrival through imaging and treatment initiation.

This study aimed to evaluate the effectiveness of this protocol in improving treatment duration and neurological outcomes in patients with AIS. We hypothesized that the early involvement of interventional neuroradiologists would accelerate treatment initiation and improve patient outcomes compared to conventional management strategies.

## 2 Materials and methods

### 2.1 Study design and setting

This retrospective study was conducted at a single stroke center located in the Tohoku region of Japan. The hospital is an emergency medical center equipped with advanced pre-hospital care capabilities, including a physician-staffed emergency vehicle and an air ambulance service. At our institution, all decisions regarding tPA administration and EVT eligibility are made exclusively by interventional neuroradiologists—physicians whose individual backgrounds include neurosurgery, neurology, or emergency medicine—while general neurologists and radiologists do not participate in treatment decision-making.

Interventional neuroradiologists operate on an on-call basis. In the conventional workflow, emergency physicians obtained imaging upon patient arrival and, based on the clinical history and imaging findings, called the on-call interventional neuroradiologist to determine tPA and EVT eligibility. In 2015, a new protocol was implemented to minimize delays by involving interventional neuroradiologists earlier. Under this protocol, emergency physicians activate the stroke team by calling interventional neuroradiologists based on pre-hospital information; however, not all eligible cases triggered protocol activation, and some patients continued to follow the conventional workflow, in which neuroradiologists were summoned only after imaging. The stroke team was not activated if those interventionists were engaged in other procedures or if the ER physician suspected a non-stroke diagnosis. Once activated, at least two interventional neuroradiologists (including trainees) convened before the patient’s arrival to participate in the initial assessment.

**Figure 1:**
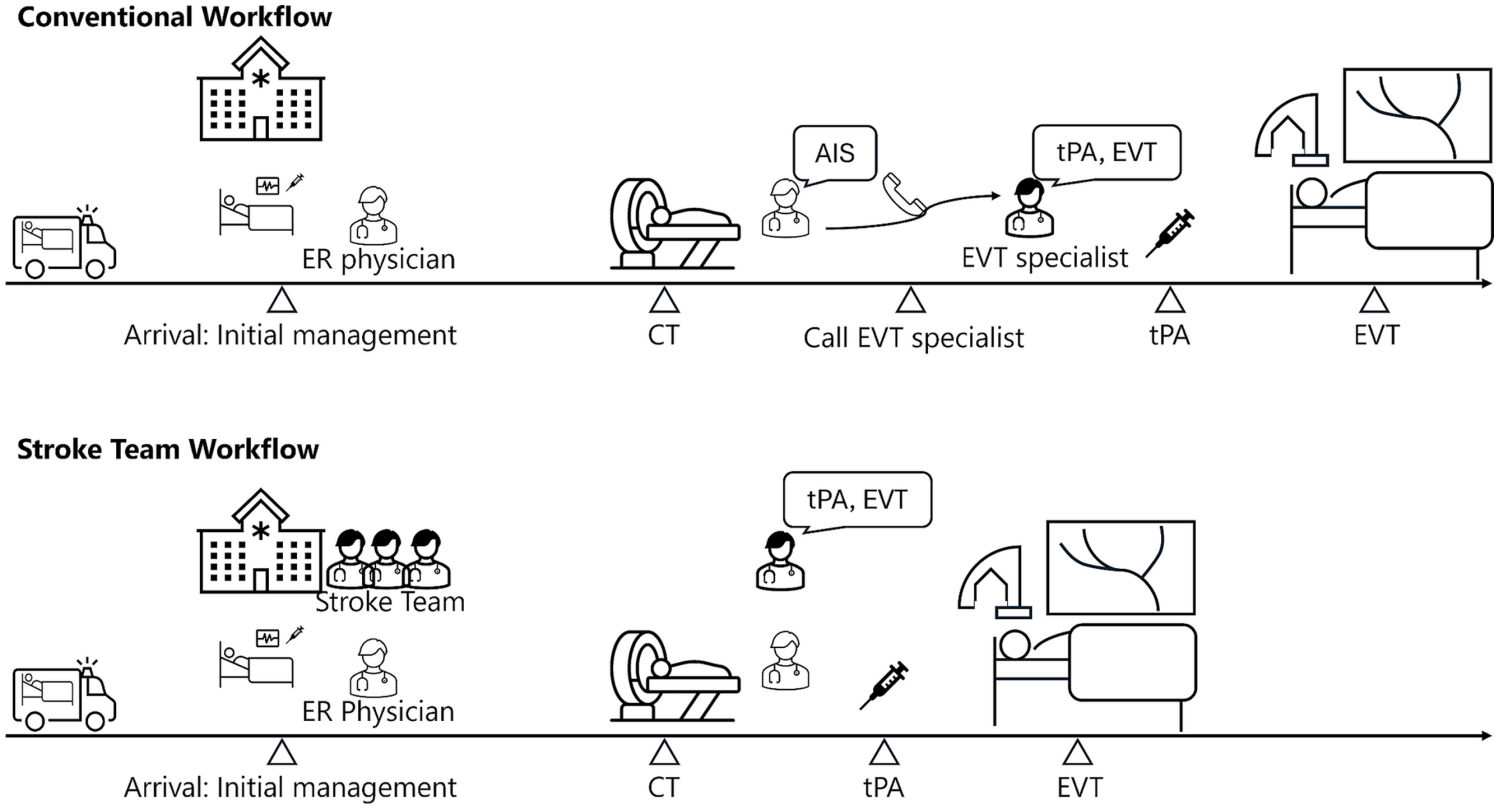
Comparison of conventional and stroke team workflow for AIS. This figure compares workflows for AIS treatment under conventional and stroke team protocols. In the conventional workflow, the emergency room (ER) physicians manage initial care and imaging before summoning an interventional neuroradiologist for EVT. The stroke team protocol positions the neuroradiologist in the ER from the outset, enabling immediate initiation of tPA administration and EVT after imaging. CT, computed tomography; AIS, acute ischemic stroke; tPA, tissue plasminogen activator; EVT, endovascular therapy.

Patients qualify for activation if they exhibited suspected stroke symptoms within 24 h of the last known well time and had a National Institutes of Health Stroke Scale (NIHSS) score of ≥3 (or were presumed to meet this threshold), as determined by information provided by paramedics or pre-hospital care physicians. Once convened, the stroke team immediately commences assessment and care upon arrival (Figure 1). Rapid imaging diagnostics, including non-contrast head CT with perfusion imaging or head MRI, are performed to confirm the diagnosis of AIS (14). The imaging results are immediately reviewed by interventional neuroradiologists, who initiate tPA administration or EVT as appropriate.

EVT was performed on patients with occlusions of the internal carotid artery, M1 or M2 segments of the middle cerebral artery, or the basilar artery, provided they exhibited a relatively large penumbra compared to the infarct core. All endovascular procedures were performed by interventional neuroradiologists. This study was approved by the Ethics Committee of Hachinohe City Hospital (Approval Number: 2425), and the requirement for informed consent was waived due to its retrospective design.

### 2.2 Patient selection and grouping

This study included (between January 2010 and December 2022) patients with AIS who were directly transported from pre-hospital locations to our ER by emergency medical service personnel and received tPA or EVT. Only those with a confirmed electronic medical record diagnosis of cerebral infarction were enrolled; cases recorded under other diagnoses were not included. Then, patients who developed AIS during their hospital stay, those transferred from other hospitals or clinics, or had incomplete data regarding outcomes or confounding variables were excluded.

Protocol activation followed specific criteria. Eligibility for activation required an estimated NIHSS score of ≥3, indicating a high probability of stroke. Protocol activation did not occur under certain circumstances, such as the unavailability of a neuroendovascular interventionist due to concurrent procedures or when the stroke was not initially suspected, as in cases of altered consciousness. Additionally, during the early implementation phase, protocol activation was occasionally missed due to limited awareness among staff.

The study population was divided into two groups: the protocol group, where patients received continuous care from initial evaluation to tPA administration or EVT under the stroke team’s management, including neuroendovascular interventionists, and the conventional group, where the stroke team was not activated, and standard treatment protocols were followed.

### 2.3 Data collection

Data were collected from electronic medical records and included demographic information such as age, sex, and modified Rankin Scale (mRS) scores (15, 16). Past medical histories, including hypertension and atrial fibrillation, were recorded. Stroke onset details were documented, including the location of onset and onset-to-door time (13, 17), defined as the duration from symptom onset to hospital arrival. For patients with uncertain onset time, the last known asymptomatic time was considered as the onset time (18). Information about pre-hospital medical care was also collected (19–21).

At admission, NIHSS scores (22) and Alberta Stroke Program Early Computed Tomography Scores (ASPECTS) (23) were recorded, along with the AIS etiology (e.g., atrial fibrillation, atherothrombotic, left-to-right shunt, or cryptogenic) and culprit lesion location (e.g., M1, M2, or M3 segment of the middle cerebral artery, internal carotid artery, basilar artery, anterior cerebral artery, or other arteries).

For treatment-related data, door-to-imaging time (time from hospital arrival to CT imaging) and door-to-needle time (only for tPA administration, representing the time from hospital arrival to tPA administration) were recorded. For patients undergoing EVT, door-to-puncture time (time from hospital arrival to arterial puncture) and door-to-recanalization time (time from hospital arrival to recanalization) were documented. The Thrombolysis in Cerebral Infarction (TICI) grade for patients undergoing EVT (24) and the occurrence of symptomatic intracranial hemorrhage or parenchymal hemorrhage without associated symptoms post-procedure were also recorded.

Neurological outcomes were assessed using mRS scores at discharge. For cases without explicit mRS scores, comprehensive evaluations were conducted based on records from physicians, nurses, and rehabilitation staff. Given that patients with a favorable prognosis often did not return for post-discharge outpatient follow-up (resulting in incomplete data on 3-month mRS scores), the primary outcome was defined as a favorable neurological outcome (mRS 0–2) at discharge.

### 2.4 Outcome measures

The primary outcome was a favorable neurological outcome at discharge, defined as an mRS score of 0–2. Secondary outcomes included door-to-imaging time, door-to-needle time, door-to-puncture time, and door-to-recanalization time as direct measures of time reduction with the stroke team, in addition to mRS distribution at discharge.

### 2.5 Statistical analysis

Descriptive statistics were calculated for all variables of interest. Continuous variables were presented as medians and interquartile ranges (IQRs), whereas categorical variables were expressed as counts and percentages. Univariate analyses were performed using the Mann–Whitney U test for continuous variables and the chi-squared test or Fisher’s exact test for categorical variables, as appropriate.

Logistic regression was employed to evaluate the primary outcome—favorable neurological outcome at discharge (mRS 0–2)—with the use of the stroke team protocol as the primary predictor. The model was adjusted for potential confounders, including age, sex, pre-hospital care, good pre-onset mRS (defined as mRS 0–2), initial NIHSS score, and onset-to-arrival time.

To assess the robustness of these findings, a series of sensitivity analyses was conducted in which the univariable logistic regression was first repeated in a 1:1 propensity-score–matched cohort—patients were matched on a set of covariates, including those used in the logistic regression model; a multivariable logistic regression was then conducted in that matched cohort, and finally the same multivariable analysis was restricted to the subgroup of patients treated after the stroke-team protocol was implemented (2015–2022; *n* = 383).

For secondary outcomes, including door-to-imaging time, door-to-needle time, door-to-puncture time, and door-to-recanalization time, multiple linear regression analyses were conducted using the same independent variables.

Additionally, the overall distribution of mRS scores at discharge (0–6) was analyzed by creating a detailed table and conducting a chi-squared test to compare the distributions between the protocol and conventional groups.

All statistical analyses were performed using Python (version 3.11.9) with the stats-models library (version 0.14.2). Before analysis, missing or infinite data values were excluded. Statistical significance was set at *p* < 0.05.

## 3 Results

### 3.1 Patient enrollment and grouping of eligible patients

**Figure 2:**
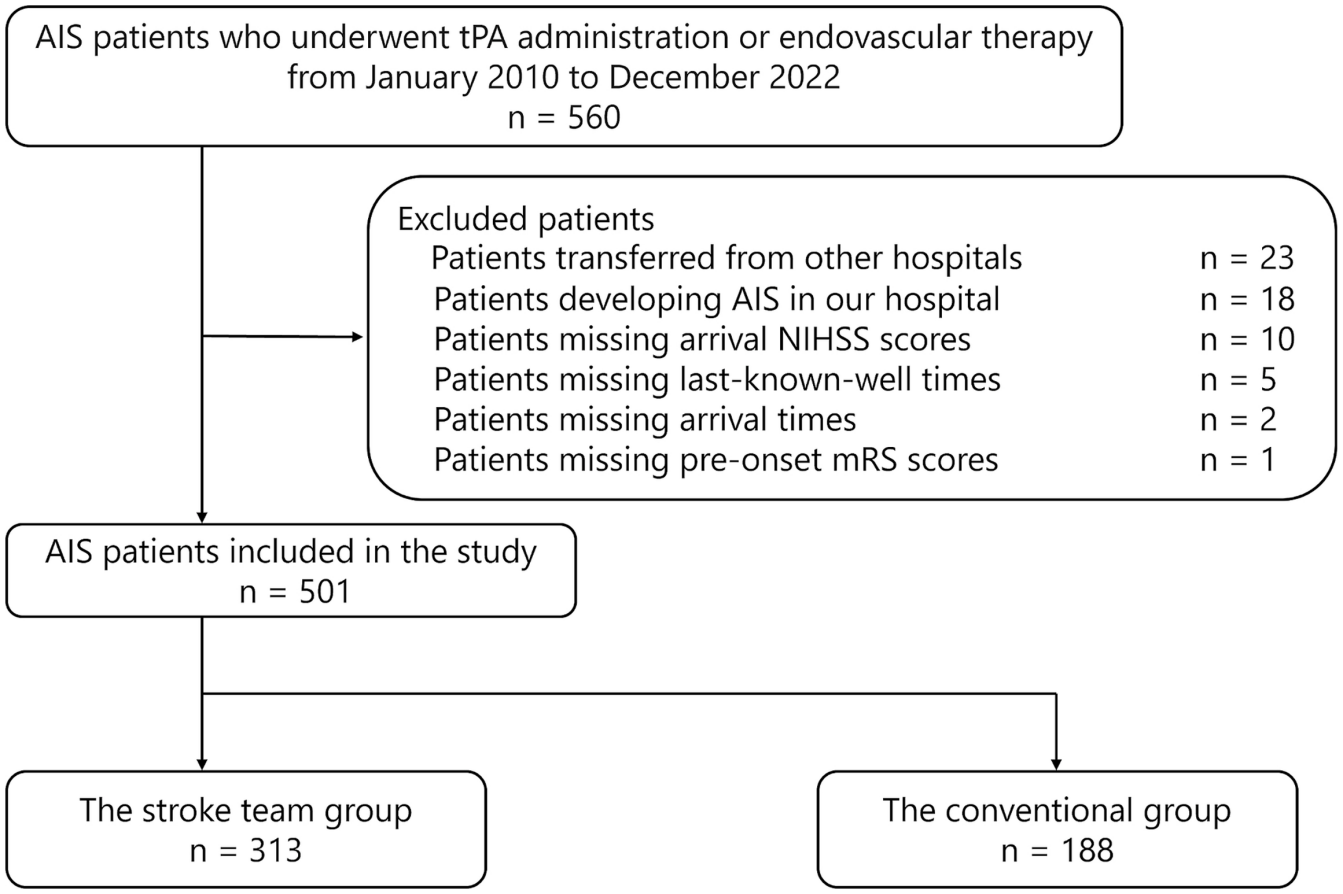
Flowchart of patient selection, summarizing exclusions and final cohort size. AIS, acute ischemic stroke; tPA, tissue plasminogen activator; NIHSS, National Institutes of Health Stroke Scale; mRS, modified Rankin Scale.

During the study period, 560 patients with AIS underwent tPA administration or EVT. Among these, 23 patients (4.1%) were transferred from other hospitals, and 18 (3.2%) developed AIS in our hospital. Overall, 519 patients who were directly transported from pre-hospital locations to the ER and received tPA or EVT were initially considered and were divided into the protocol and conventional groups. After excluding 10 patients with unknown NIHSS scores at admission, five with unknown last-known-well time, two with unknown hospital arrival time, and one with unknown pre-onset mRS scores, 501 patients were deemed eligible for primary analysis (313 patients in the protocol group and 188 in the conventional group) (Figure 2). The excluded cases represented less than 5% of the total cohort, enabling a complete-case analysis approach consistent with prior methodologies (25).

### 3.2 Patients’ characteristics and missing data

Baseline characteristics of the 501 patients included in the primary analysis were comprehensively analyzed (Table 1). Missing values for baseline variables, such as past medical histories (*n* = 16), location of onset (*n* = 2), etiology (*n* = 13), and ASPECTS (*n* = 1), were reported. However, these variables were not considered confounding factors in the primary analysis, and their missing values did not exclude patients.

**Table 1:** Pre-hospital and in-hospital characteristics: stroke team workflow vs. conventional workflow.

	Overall	Conventional workflow	Stroke team workflow	*p*-value
Variables	(*n* = 501)	(*n* = 188)	(*n* = 313)	
Prehospital variables				
Age (years)	78.0 (70.0–85.0)	77.0 (70.0–83.2)	78.0 (71.0–85.0)	0.102
Male, *n* (%)	274 (54.7)	93 (49.5)	181 (57.8)	0.084
Good pre-onset mRS, *n* (%)	456 (91.0)	176 (93.6)	280 (89.5)	0.157
Onset in the nearest medical service area, *n* (%)	335 (67.1)	139 (74.7)	196 (62.6)	0.007
Presence of prehospital medical care, *n* (%)	358 (71.5)	101 (53.7)	257 (82.1)	<0.002
Onset to arrival time (min)	84.0 (51.0–177.0)	65.5 (44.0–125.8)	101.0 (57.0–206.0)	<0.001
Atrial fibrillation, *n* (%)	68 (13.6)	31 (16.5)	37 (11.8)	0.179
Hypertension, *n* (%)	82 (16.4)	30 (16.0)	52 (16.6)	0.946
In-hospital variables				
Arrival NIHSS (points)	16.0 (8.0–23.0)	15.0 (6.0–23.0)	17.0 (10.0–23.0)	0.018
ASPECT (points)	10.0 (9.0–10.0)	10.0 (9.0–10.0)	10.0 (9.0–10.0)	0.003
Suspected embolic stroke, *n* (%)	354 (72.5)	131 (71.6)	223 (73.1)	0.793
Administration of tPA, *n* (%)	401 (80.0)	172 (91.5)	229 (73.2)	<0.001
Endovascular therapy, *n* (%)	261 (52.1)	32 (17.0)	229 (73.2)	<0.001
Culprit lesions, *n* (%)				
M1	135 (26.9)	48 (25.5)	87 (27.8)	<0.001
M2	116 (23.2)	40 (21.3)	76 (24.3)	
M3	2 (0.4)	1 (0.5)	1 (0.3)	
ICA	102 (20.4)	24 (12.8)	78 (24.9)	
ACA	8 (1.6)	4 (2.1)	4 (1.3)	
BA	19 (3.8)	9 (4.8)	10 (3.2)	
Others	76 (15.2)	50 (26.6)	26 (8.3)	
Good recanalization, *n* (%)	211 (74.8)	23 (76.7)	188 (74.6)	0.981
Symptomatic ICH, *n* (%)	45 (9.1)	4 (2.1)	41 (13.3)	<0.001
parenchymal hemorrhage without associated symptoms, *n* (%)	38 (7.7)	16 (8.6)	22 (7.1)	0.69

Data were presented as medians (interquartile ranges) for continuous variables and numbers (proportions) for categorical values. Proportions excluded missing data. Good mRS was defined as a modified Rankin Scale (mRS) score of 0–2, and good recanalization was defined as Thrombolysis in Cerebral Infarction (TICI) grades of 3, 2c, or 2b. mRS, modified Rankin Scale; NIHSS, National Institutes of Health Stroke Scale; ASPECTS, Alberta Stroke Program Early Computed Tomography Score; tPA, tissue plasminogen activator; M1, first segment of the middle cerebral artery; M2, second segment of the middle cerebral artery; M3, third segment of the middle cerebral artery; ICA, internal carotid artery; ACA, anterior cerebral artery; BA, basilar artery; TICI, thrombolysis in cerebral Infarction; ICH, intracerebral hemorrhage.

For secondary outcome analyses, cases with missing values specific to the secondary outcome variables were excluded. Among patients who received tPA, one case lacked documentation of administration time. For patients who underwent EVT, the puncture time was missing in three cases, and recanalization time was unavailable for six cases. These exclusions were applied to maintain data integrity while minimizing the potential impact of missing data on the results.

The median onset-to-door time was 84 min (IQR: 51–177), and the median NIHSS score at admission was 16 (IQR: 8–23). The most common etiology of AIS was atrial fibrillation (72.5%), and the most frequent culprit lesion was the M1 segment of the middle cerebral artery (26.9%). In the protocol group, 229 patients (73.2%) received tPA compared to 172 (91.5%) in the conventional group, whereas EVT was administered to 229 patients (73.2%) in the protocol group and 32 (17.0%) in the conventional group.

### 3.3 Patients’ outcomes

**Figure 3:**
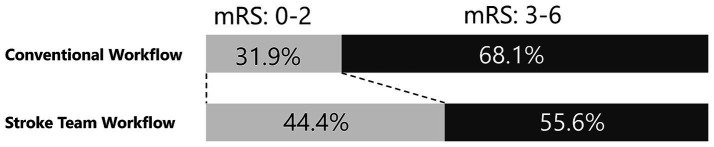
Adjusted neurological outcome. This figure shows the proportions of favorable neurological outcomes at discharge for the stroke team protocol and conventional workflows. The stroke team group had a higher proportion of favorable outcomes (44.4% vs. 31.9%), with an adjusted odds ratio of 2.92 (95% confidence interval: 1.83–4.66, *p* < 0.001). A favorable neurological outcome was defined as a modified Rankin Scale (mRS) score of 0–2.

The primary outcome, a favorable neurological outcome (mRS 0–2) at discharge, was achieved in 139 (44.4%) patients in the protocol group and 60 (31.9%) in the conventional group (Figure 3; Table 2). After adjusting for age, sex, presence of pre-hospital medical care, initial NIHSS score, onset-to-arrival time, and pre-onset mRS, the protocol group remained significantly more likely to achieve a favorable outcome (adjusted OR 2.92; 95% CI 1.83–4.66; *p* < 0.001) (Table 3). In the propensity-score–matched cohort (using the same covariates for matching), univariable analysis yielded OR 2.17 (95% CI 1.35–3.49; *p* = 0.002), and multivariable analysis yielded adjusted OR 3.45 (95% CI 1.94–6.15; *p* < 0.001). Finally, in a sensitivity analysis restricted to patients treated after protocol implementation (2015–2022; *n* = 383), multivariable logistic regression produced an adjusted OR of 2.75 (95% CI 1.44–5.27; *p* = 0.002), confirming the robustness of the association.

**Table 2:** Neurological outcomes: stroke team workflow vs. conventional workflow.

	Conventional workflow	Stroke team workflow	*p*-value
Variables	(*n* = 188)	(*n* = 313)	
Good mRS at discharge	60 (31.9)	139 (44.4)	0.006
mRS at discharge			
0: No symptoms at all	8 (4.3)	26 (8.3)	0.014
1: No significant disability despite symptoms	20 (10.6)	41 (13.1)	
2: Slight disability	32 (17.0)	72 (23.0)	
3: Moderate disability	35 (18.6)	40 (12.8)	
4: Moderately severe disability	35 (18.6)	63 (20.1)	
5: Severe disability	34 (18.1)	54 (17.3)	
6: Dead	24 (12.8)	17 (5.4)	

Data were presented as numbers (percentages). A good modified Rankin Scale (mRS) score was defined as a score of 0–2. mRS, modified Rankin Scale.

**Table 3:** Stroke team workflow effects: univariate and multivariate analyses.

Analysis type	Odds ratio	95% CI	*p*-value
Univariable analysis	1.7	1.17–2.49	0.006
Multivariable analysis	2.92	1.83–4.66	<0.001

CI, confidence interval.

**Figure 4:**
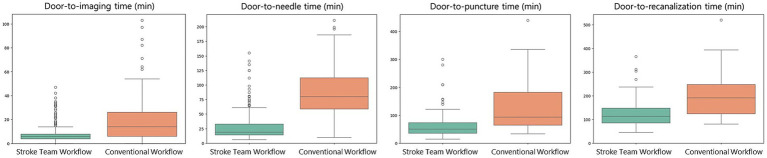
Adjusted time-based outcomes. The stroke team protocol reduced treatment times, as indicated by the adjusted regression coefficients: door-to-imaging time (−8.3 min, 95% confidence interval [CI]: −10.5−−6.1, *p* < 0.001), door-to-needle time (−55.9 min, 95% CI: −62.7−−49.1, *p* < 0.001), door-to-puncture time (−59.8 min, 95% CI: −77.2−−42.5, *p* < 0.001), and door-to-recanalization time (−73.7 min, 95% CI: −97.1−−50.4, *p* < 0.001).

Regarding secondary outcomes, the stroke team workflow was associated with substantially shorter treatment times compared to the conventional workflow. In the conventional group, the median (IQR) times were 14.0 min (6.0–26.2) for door-to-imaging time, 80.0 min (58.8–112.2) for door-to-needle time, 94.0 min (64.2–182.5) for door-to-puncture time, and 192.0 min (124.0–248.0) for door-to-recanalization time. In the protocol group, the corresponding times were 6.0 min (4.0–8.0), 19.0 min (14.0–33.0), 51.0 min (36.0–74.0), and 114.0 min (85.2–148.5), respectively (Table 4). Adjusted regression coefficients further demonstrated the significant reductions in treatment times associated with the protocol: door-to-imaging time (−8.3 min, 95% CI: −10.5−−6.1, *p* < 0.001), door-to-needle time (−55.9 min, 95% CI: −62.7−−49.1, *p* < 0.001), door-to-puncture time (−59.8 min, 95% CI: −77.2−−42.5, *p* < 0.001), and door-to-recanalization time (−73.7 min, 95% CI: −97.1−−50.4, *p* < 0.001) (Figure 4; Table 5).

**Table 4:** Time-based outcomes: door-to-imaging, door-to-needle, door-to-puncture, and door-to-recanalization times.

Time metrics	Conventional workflow	Stroke team workflow	*p*-value
Door-to-imaging time (min)	14.0 (6.0–26.2)	6.0 (4.0–8.0)	<0.001
Door-to-needle time (min)	80.0 (58.8–112.2)	19.0 (14.0–33.0)	<0.001
Door-to-puncture time (min)	94.0 (64.2–182.5)	51.0 (36.0–74.0)	<0.001
Door-to-recanalization time (min)	192.0 (124.0–248.0)	114.0 (85.2–148.5)	<0.001

Data was presented as medians (interquartile ranges).

**Table 5:** Adjusted effects of stroke team workflow on time metrics.

Time metrics	Regression coefficient	95% CI	*p*-value
Door-to-imaging time (min)	−8.3	−10.5−−6.1	<0.001
Door-to-needle time (min)	−55.9	−62.7−−49.1	<0.001
Door-to-puncture time (min)	−59.8	−77.2−−42.5	<0.001
Door-to-recanalization time (min)	−73.7	−97.1−−50.4	<0.001

CI, confidence interval.

## 4 Discussion

This retrospective study evaluated the impact of the early involvement of neuroendovascular specialists in the ER on the management of patients with AIS undergoing tPA administration or EVT. The findings demonstrated that implementing a stroke team management protocol significantly improved the proportion of patients achieving favorable neurological outcomes. Additionally, the protocol substantially reduced treatment times, including door-to-needle time, door-to-puncture time, and door-to-recanalization time, compared to the conventional approach. The significant improvements observed with the stroke team management underscore the potential of this novel system to streamline the care pathways for patients with AIS.

Time is a critical factor in AIS management (26). Optimizing in-hospital workflows to reduce the time from patient arrival to treatment has been shown to enhance patient outcomes (2–5). Despite these efforts, delays often arise because interventional neuroradiologists are typically consulted only after image acquisition (27, 28). A study has suggested that involving interventional neuroradiologists early in the AIS care pathway, starting from the patient’s initial presentation may reduce door-to-puncture times (29). A proposed strategy to achieve faster intervention and improve neurological outcomes involves directing selected patients with LVO in the early time window directly to the angiography suite (30). However, this direct-to-angiography (DTA) approach presents several challenges. Firstly, the limited diagnostic accuracy of current pre-hospital tools in distinguishing AIS from stroke mimics, such as seizures or migraines, may result in unnecessary activation of the neuro-angiography suite. Secondly, many DTA setups lacked advanced perfusion imaging capabilities within the angiography suite, which are essential for assessing infarct burden or mismatches in late-window thrombectomy cases. Additionally, false-positive detection of LVOs, such as cases with recanalized vessels, may lead to unnecessary utilization of the angiography suite, delaying treatment for other eligible patients (31, 32). To address these limitations, a new protocol was developed at our institution to involve interventional neuroendovascular specialists in the initial patient assessment. This approach aimed to reduce delays that could compromise treatment efficacy by enabling earlier specialist consultation and streamlining the mobilization of the endovascular team. Additionally, the protocol provided contingency measures, such as arranging transfers to other facilities (33) when prolonged suite unavailability was anticipated and avoiding unnecessary suite use, a notable drawback of the DTA approach. By ensuring the immediate involvement of neuroendovascular specialists upon patient presentation, the protocol facilitated rapid imaging interpretation and expedited treatment decisions. These findings underscore the importance of a comprehensive approach to AIS management. Further research is needed to quantify the benefits of this integrated approach and to explore its feasibility across diverse healthcare settings. Nonetheless, this study’s findings suggest that optimizing both the care delivery environment and human resource factors holds promise for improving AIS management.

While these findings support a potential causal link between early neuro-interventionalist involvement and improved outcomes, the observational design of this study precludes definitive causal inference. To mitigate confounding, known covariates in multivariable models were adjusted for, performing propensity-score matching on the same set of predictors and restricting sensitivity analysis to patients treated after protocol implementation, thereby demonstrating the robustness of these results. Nevertheless, the possibility of residual confounding by unmeasured factors cannot be entirely excluded. Moreover, during the study period, advancements in EVT devices and the expansion of EVT eligibility time windows may also have contributed to the observed improvements in outcomes. Protocol adherence among eligible patients was approximately 10% in the first year but gradually increased thereafter, surpassing 80% by the third year and maintaining levels above 80% thereafter, and this variable adherence rate may have influenced the results. Even so, the observed improvements in time metrics and clinical outcomes are biologically plausible given the well-established time-dependent nature of stroke interventions (2–6) and the critical role of rapid decision-making in acute stroke care.

The generalizability of these findings should be considered in light of the specific characteristics and resources of our hospital. Although our protocol demonstrated clear benefits in our stroke center, its activation threshold of NIHSS score of ≥3, applied only to patients with a confirmed diagnosis of AIS, may nonetheless mobilize neuro-interventionalists for a variety of non-cerebrovascular conditions that present with similar deficits, potentially straining limited human and material resources. Smaller hospitals or those with less ready access to neurointerventional expertise may need to adapt both the workflow and the activation criteria to their local constraints (34). Future work should, therefore, aim not only to refine and validate stroke-specific activation thresholds that optimize sensitivity and specificity but also to assess the feasibility and effectiveness of similar protocols across diverse healthcare settings, including community hospitals and rural areas.

This study had several limitations. Firstly, instances of suboptimal stroke team activation occurred, particularly during the early implementation phase of the protocol. These cases included situations where the protocol was not activated despite meeting the criteria due to insufficient awareness, cases where the neuro-interventionalist was unavailable due to ongoing surgeries, and cases where the stroke was not initially suspected due to presenting symptoms such as altered consciousness. These challenges highlight the importance of continuous education and refinement of activation criteria. Secondly, incomplete baseline information may have introduced confounding. Medical history and other key variables were collected shortly after admission and were sometimes missing or inaccurate, potentially affecting model adjustment and result interpretation. Thirdly, several methodological constraints inherent in retrospective cohort studies must be considered (35). Selection bias, unmeasured confounders, and missing data—particularly in the variables mentioned above—may have influenced the results despite multivariable adjustment, propensity-score matching, and other sensitivity analyses. Fourthly, temporal and clinical sources of bias remain. The stroke-team protocol was introduced in 2015, and its adherence rose from ≈10% in the first year to >80% after 2017, coinciding with progressive improvements in EVT devices and peri-procedural care; thus, patients in the protocol group disproportionately benefited from more recent treatment options. Furthermore, the decision not to activate the stroke team often reflected clinical considerations (e.g., presumed stroke mimics and interventionalist unavailability). Consequently, the two groups differed in both measured and unmeasured ways, which may have biased group allocation even after statistical adjustment. Lastly, the absolute sample size was modest (501 recanalization cases over 13 years), limiting statistical power for subgroup analyses and the precision of effect estimates; therefore, the present findings should be interpreted with caution and validated in larger multi-center cohorts. In conclusion, early involvement of neuro-interventionalists was associated with faster treatment initiation and a higher likelihood of favorable functional status at discharge in this retrospective cohort. Although implementing such protocols requires substantial resources and training, this approach may offer a practical means of streamlining modern stroke care. However, residual confounding and temporal improvements in therapy limit causal inference; prospective multi-center studies are warranted to confirm these observations and to explore their generalizability across diverse healthcare settings in the era of expanding EVT.

## Data Availability

The raw data supporting the conclusions of this article will be made available by the authors, without undue reservation.
