# Supplementary material for: Effect of early neuroendovascular team involvement in acute stroke protocol: a retrospective study
Source: Front Neurol. 2025 Jun 17;16:1568572. doi: 10.3389/fneur.2025.1568572 (PMC12209294; doi:10.3389/fneur.2025.1568572)
Supplement: Supplementary file 1 [file Supplementary_file_1.docx]

Table S1. Prehospital and in-hospital variables of stroke team workflow and conventional workflow groups before and after propensity score matching.

|  | Before Propensity Score Matching | | |  | After Propensity Score Matching | | |
| --- | --- | --- | --- | --- | --- | --- | --- |
| Continuous variables | Stroke team workflow | Conventional workflow | SMD |  | Stroke team workflow | Conventional workflow | SMD |
| Age (years) | 78.5 (71.0–85.0) | 77.0 (70.0–84.0) | 0.13 |  | 78.0 (70.0–86.0) | 78.0 (70.0–84.0) | 0.10 |
| Onset to arrival time (min) | 98.5 (56.8–206.0) | 66.0 (44.0–125.0) | 0.42 |  | 97.0 (59.0–199.0) | 67.0 (45.0–125.0) | 0.45 |
| Arrival NIHSS (points) | 17.0 (10.0–23.0) | 15.0 (6.0–22.0) | 0.21 |  | 15.0 (8.0–21.0) | 15.0 (7.0–23.0) | -0.02 |
| ASPECTS (points) | 10.0 (8.8–10.0) | 10.0 (9.0–10.0) | -0.30 |  | 10.0 (9.0–10.0) | 10.0 (9.0–10.0) | 0.07 |
|  |  |  |  |  |  |  |  |
| Categorical variables | Stroke team workflow | Conventional workflow | SD |  | Stroke team workflow | Conventional workflow | SD |
| Male, n (%) | 177 (58.2%) | 88 (48.6%) | 0.19 |  | 75 (50.3%) | 83 (55.7%) | -0.11 |
| Good pre-onset mRS, n (%) | 1.0 (1.0–1.0) | 1.0 (1.0–1.0) | -0.15 |  | 1.0 (1.0–1.0) | 1.0 (1.0–1.0) | -0.09 |
| Onset in the nearest medical service area, n (%) | 192 (63.2%) | 138 (76.2%) | -0.29 |  | 101 (67.8%) | 109 (73.2%) | -0.12 |
| Presence of prehospital medical care, n (%) | 249 (81.9%) | 97 (53.6%) | 0.64 |  | 97 (65.1%) | 97 (65.1%) | 0.00 |
| Atrial fibrillation, n (%) | 36 (11.8%) | 31 (17.1%) | -0.15 |  | 17 (11.4%) | 20 (13.4%) | -0.06 |
| Hypertension, n (%) | 51 (16.8%) | 27 (14.9%) | 0.05 |  | 31 (20.8%) | 24 (16.1%) | 0.12 |
| Suspected embolic stroke, n (%) | 222 (73.0%) | 130 (71.8%) | 0.03 |  | 110 (73.8%) | 106 (71.1%) | 0.06 |

Data were presented as medians (interquartile ranges) for continuous variables and numbers (proportions) for categorical values. Proportions excluded missing data.

Good mRS was defined as a modified Rankin Scale (mRS) score of 0–2.

mRS, modified Rankin Scale; NIHSS, National Institutes of Health Stroke Scale; ASPECTS, Alberta Stroke Program Early Computed Tomography Score.

Table S2. Stroke team workflow effects: univariate and multivariate analyses after propensity score matching.

| Analysis type | Odds ratio | 95% CI | *p*-value |
| --- | --- | --- | --- |
| Univariable analysis | 2.17 | 1.35–3.49 | 0.002 |
| Multivariable analysis | 3.45 | 1.94–6.15 | <0.001 |

CI, confidence interval.

Table S3. Stroke team workflow effects: multivariate analyses restricted to data from January 2015 onward.

| Analysis type | Odds ratio | 95% CI | *p*-value |
| --- | --- | --- | --- |
| Multivariable analysis | 2.75 | 1.44–5.27 | 0.002 |

CI, confidence interval.
