# Supplementary material for: Effect of early neuroendovascular team involvement in acute stroke protocol: a retrospective study
Source: Front Neurol. 2025 Jun 17;16:1568572. doi: 10.3389/fneur.2025.1568572 (PMC12209294; doi:10.3389/fneur.2025.1568572)
Supplement: Supplementary file 2 [file Table_1.docx]

STROBE Statement—checklist of items that should be included in reports of observational studies

|  | Item No. | Recommendation | Page  No. | Relevant text from manuscript |
| --- | --- | --- | --- | --- |
| **Title and abstract** | 1 | (*a*) Indicate the study’s design with a commonly used term in the title or the abstract |  | ・This single-center retrospective observational study |
|  |  | (*b*) Provide in the abstract an informative and balanced summary of what was done and what was found |  | ・ patients with AIS transported to our emergency room (ER) who received tPA or EVT between January 2010 and December 2022.  ・Favorable neurological outcomes at discharge (mRS 0–2) were significantly more frequent in the protocol group than in the conventional group |
| Introduction | | | |  |
| Background/rationale | 2 | Explain the scientific background and rationale for the investigation being reported |  | ・Acute ischemic stroke (AIS) caused by major vessel occlusion… |
| Objectives | 3 | State specific objectives, including any prespecified hypotheses |  | ・This study aimed to evaluate the effectiveness of this protocol… |
| Methods | | | |  |
| Study design | 4 | Present key elements of study design early in the paper |  | ・This retrospective study |
| Setting | 5 | Describe the setting, locations, and relevant dates, including periods of recruitment, exposure, follow-up, and data collection |  | ・a single stroke center located in the Tohoku region… |
| Participants | 6 | (*a*) *Cohort study*—Give the eligibility criteria, and the sources and methods of selection of participants. Describe methods of follow-up  *Case-control study*—Give the eligibility criteria, and the sources and methods of case ascertainment and control selection. Give the rationale for the choice of cases and controls  *Cross-sectional study*—Give the eligibility criteria, and the sources and methods of selection of participants |  | ・This study included patients… |
|  |  | (*b*) *Cohort study*—For matched studies, give matching criteria and number of exposed and unexposed  *Case-control study*—For matched studies, give matching criteria and the number of controls per case |  | NA |
| Variables | 7 | Clearly define all outcomes, exposures, predictors, potential confounders, and effect modifiers. Give diagnostic criteria, if applicable |  | ・Logistic regression was employed to evaluate the primary outcome… |
| Data sources/ measurement | 8* | For each variable of interest, give sources of data and details of methods of assessment (measurement). Describe comparability of assessment methods if there is more than one group |  | ・Data collection  Data were collected from electronic medical records and… |
| Bias | 9 | Describe any efforts to address potential sources of bias |  | ・Logistic regression was employed to evaluate… |
| Study size | 10 | Explain how the study size was arrived at |  | NA |

Continued on next page

| Quantitative variables | 11 | Explain how quantitative variables were handled in the analyses. If applicable, describe which groupings were chosen and why |  | ・Descriptive statistics were calculated… |
| --- | --- | --- | --- | --- |
| Statistical methods | 12 | (*a*) Describe all statistical methods, including those used to control for confounding |  | ・Descriptive statistics were calculated… |
|  |  | (*b*) Describe any methods used to examine subgroups and interactions |  | NA |
|  |  | (*c*) Explain how missing data were addressed |  | ・After excluding 10 patients with unknown… |
|  |  | (*d*) *Cohort study*—If applicable, explain how loss to follow-up was addressed  *Case-control study*—If applicable, explain how matching of cases and controls was addressed  *Cross-sectional study*—If applicable, describe analytical methods taking account of sampling strategy |  | ・To assess the robustness of these findings… |
|  |  | (*e*) Describe any sensitivity analyses |  | NA |
| Results | | | | |
| Participants | 13* | (a) Report numbers of individuals at each stage of study—eg numbers potentially eligible, examined for eligibility, confirmed eligible, included in the study, completing follow-up, and analysed |  | ・Patient **enrollment** and **grouping** of **eligible patients…** |
|  |  | (b) Give reasons for non-participation at each stage |  | ・ During the study period, 560 patients with AIS… |
|  |  | (c) Consider use of a flow diagram |  | Figure 2 |
| Descriptive data | 14* | (a) Give characteristics of study participants (eg demographic, clinical, social) and information on exposures and potential confounders |  | Table 1 |
|  |  | (b) Indicate number of participants with missing data for each variable of interest |  | ・After excluding 10 patients with unknown… |
|  |  | (c) *Cohort study*—Summarise follow-up time (eg, average and total amount) |  | NA |
| Outcome data | 15* | *Cohort study*—Report numbers of outcome events or summary measures over time |  | ・Patients’ outcomes… |
|  |  | *Case-control study—*Report numbers in each exposure category, or summary measures of exposure |  |  |
|  |  | *Cross-sectional study—*Report numbers of outcome events or summary measures |  |  |
| Main results | 16 | (*a*) Give unadjusted estimates and, if applicable, confounder-adjusted estimates and their precision (eg, 95% confidence interval). Make clear which confounders were adjusted for and why they were included |  | ・Logistic regression was employed to evaluate…  Line 188-194:  Patients’ outcomes… |
|  |  | (*b*) Report category boundaries when continuous variables were categorized |  | NA |
|  |  | (*c*) If relevant, consider translating estimates of relative risk into absolute risk for a meaningful time period |  | NA |

Continued on next page

| Other analyses | 17 | Report other analyses done—eg analyses of subgroups and interactions, and sensitivity analyses |  | ・Univariate analyses were performed… |
| --- | --- | --- | --- | --- |
| Discussion | | | | |
| Key results | 18 | Summarise key results with reference to study objectives |  | ・The findings demonstrated that implementing a stroke team… |
| Limitations | 19 | Discuss limitations of the study, taking into account sources of potential bias or imprecision. Discuss both direction and magnitude of any potential bias |  | ・This study had several limitations… |
| Interpretation | 20 | Give a cautious overall interpretation of results considering objectives, limitations, multiplicity of analyses, results from similar studies, and other relevant evidence |  | ・Although our results suggest a causal relationship between… |
| Generalisability | 21 | Discuss the generalisability (external validity) of the study results |  | ・ The generalizability of our findings should be considered |
| Other information | |  | | |
| Funding | 22 | Give the source of funding and the role of the funders for the present study and, if applicable, for the original study on which the present article is based |  | ・Funding… |

*Give information separately for cases and controls in case-control studies and, if applicable, for exposed and unexposed groups in cohort and cross-sectional studies.

**Note:** An Explanation and Elaboration article discusses each checklist item and gives methodological background and published examples of transparent reporting. The STROBE checklist is best used in conjunction with this article (freely available on the Web sites of PLoS Medicine at http://www.plosmedicine.org/, Annals of Internal Medicine at http://www.annals.org/, and Epidemiology at http://www.epidem.com/). Information on the STROBE Initiative is available at www.strobe-statement.org.
